# Effectiveness of a Web-Based Intervention in Reducing Depression and Sickness Absence: Randomized Controlled Trial

**DOI:** 10.2196/jmir.6546

**Published:** 2017-06-15

**Authors:** Till Beiwinkel, Tabea Eißing, Nils-Torge Telle, Elisabeth Siegmund-Schultze, Wulf Rössler

**Affiliations:** ^1^ Innovation Incubator Competence Tandem Integrated Care Leuphana University of Lüneburg Lüneburg Germany; ^2^ KKH Kaufmännische Krankenkasse Abteilung Leistungs- und Versorgungsmanagement Hannover Germany; ^3^ Institute of Psychiatry Laboratory of Neuroscience (LIM 27) University of Sao Paulo Sao Paulo Brazil; ^4^ Psychiatric University Hospital Zürich University Zürich Switzerland; ^5^ Department of Psychiatry and Psychotherapy Campus Charité Mitte Charité – Universitätsmedizin Berlin Berlin Germany

**Keywords:** Internet, depression, absenteeism, cognitive therapy, randomized controlled trial

## Abstract

**Background:**

Depression is highly prevalent in the working population and is associated with significant loss of workdays; however, access to evidence-based treatment is limited.

**Objective:**

This study evaluated the effectiveness of a Web-based intervention in reducing mild to moderate depression and sickness absence.

**Methods:**

In an open-label randomized controlled trial, participants were recruited from a large-scale statutory health insurance and were assigned to two groups. The intervention group had access to a 12 week Web-based program consisting of structured interactive sessions and therapist support upon request. The wait-list control group had access to unguided Web-based psycho-education. Depressive symptoms were self-assessed at baseline, post-treatment, and follow-up (12 weeks after treatment) using the Patient Health Questionnaire (PHQ-9) and Beck Depression Inventory (BDI-II) as primary outcome measures. Data on sickness absence was retrieved from health insurance records. Intention-to-treat (ITT) analysis and per-protocol (PP) analysis were performed.

**Results:**

Of the 180 participants who were randomized, 88 completed the post-assessment (retention rate: 48.8%, 88/180). ITT analysis showed a significant between-group difference in depressive symptoms during post-treatment in favor of the intervention group, corresponding to a moderate effect size (PHQ-9: *d*=0.55, 95% CI 0.25-0.85, *P*<.001, and BDI-II: *d*=0.41, CI 0.11-0.70, *P*=.004). PP analysis partially supported this result, but showed a non-significant effect on one primary outcome (PHQ-9: *d*=0.61, 95% CI 0.15-1.07, *P*=.04, and BDI-II: *d*=0.25 95% CI −0.18 to 0.65, *P*=.37). Analysis of clinical significance using reliable change index revealed that significantly more participants who used the Web-based intervention (63%, 63/100) responded to the treatment versus the control group (33%, 27/80; *P*<.001). The number needed to treat (NNT) was 4.08. Within both groups, there was a reduction in work absence frequency (IG: −67.23%, *P*<.001, CG: −82.61%, *P*<.001), but no statistical difference in sickness absence between groups was found (*P*=.07).

**Conclusions:**

The Web-based intervention was effective in reducing depressive symptoms among adults with sickness absence. As this trial achieved a lower power than calculated, its results should be replicated in a larger sample. Further validation of health insurance records as an outcome measure for eHealth trials is needed.

**Trial Registration:**

International Standard Randomized Controlled Trial Number (ISRCTN): 02446836; http://www.isrctn.com/ISRCTN02446836 (Archived by WebCite at http://www.webcitation.org/6jx4SObnw)

## Introduction

Depression is highly prevalent in the working population [1]. It is estimated that over the course of one year, up to 26.7% of adults experience depressive symptoms and about 8.9% fulfill all criteria for a depressive disorder [2]. The resulting impairment and functional disability poses a substantial burden for the affected individual as well as the economy. Depressed employees have higher health care costs than those without depression [3,4], which in Europe contribute to a total estimated cost of 118 billion Euros per year [5].

Depression is linked to a high loss of work days [6]. In Germany, depression is a major driver of sickness absence and produces higher durations of sickness absence than other diagnoses of mental disorders [7]. When employees return to work after a depressive episode, distress often remains and performance is reduced [8,9]. Therefore, maintaining work capacity should be an important goal of clinical interventions. However, health promotion interventions targeting occupational health in employees with depression have been developed with mixed results [10-13]. Access to treatment remains limited, and the existing personal and structural barriers prevent those affected by depression from seeking timely, evidence-based help [14-16].

Web-based interventions are a promising tool to overcome the treatment gap in depression [17]. While generally using similar techniques as face-to-face therapy, such interventions are commonly delivered through websites and allow participants to access content at any time and work through lessons at their own pace. Web-based interventions vary in the level of therapist support [18], from entirely self-help to guided formats including regular therapist contact (eg, feedback via email). The advantages of Web-based interventions are their accessibility, a low threshold for help-seeking, relative anonymity, the patients’ active role in (guided) self-help, and their low costs. However, in studies comparing Web-based interventions to usual care, risks associated with the dissemination of Web-based interventions have been reported as well [19,20]. Among the working population, Web-based interventions could especially benefit those who do not want to seek regular treatment because of negative perceptions of mental ill-health at the workplace.

The effectiveness of Web-based interventions in reducing depressive symptoms has been demonstrated repeatedly, but effect sizes vary considerably across studies [21-23]. For example, in the 19 studies that were included in the meta-analysis by Richards and Richardson [21], depression improvement in comparison with a control group ranged from no effect (*d*=−0.03) to strong effects (*d*=1.43). This heterogeneity makes it necessary to evaluate the interventions separately. Methodologically, weak control groups (eg, wait-list control instead of active control groups) and failure to employ intention-to-treat principles lead to an overestimation of the treatment effect [24]. Web-based interventions for depression have been studied among different clinical populations in Germany [25-29] but, to the best of our knowledge, no studies have yet focused on a Web-based intervention among a population with sick leave due to depression.

Participant self-reports are the primary outcome measure of eHealth trials. However, the lack of independent outcome assessments and the sole reliance on self-report measures limits this evolving field. For example, a report on the methodological quality of randomized controlled trials of Web-based interventions concluded that an increased use of independent outcome measurements is needed to improve the validity of efficacy studies [24]. To date, few studies employ independent outcomes and such attempts are limited to observer ratings of symptoms and do not extend to objective behavioral measurement of work absenteeism [30-32]. The lack of objective sickness absence measurements in research on Web-based interventions is surprising because sickness absence is frequently used as an integrated measure of health in other fields [33].

This study examined the effectiveness of a guided Web-based intervention in reducing depression and sickness absence among a high-risk population using both self-assessed depression and sickness absence assessments from health insurance records. We hypothesized that the Web-based intervention would be more effective in reducing depressive symptoms and sickness absence than the control group.

## Methods

### Study Design

This was a two-armed open-label randomized controlled trial. Participants were randomly assigned to either the intervention group (IG), with access to the guided Web-based intervention, or the wait-list control group (CG), with access to unguided Web-based psycho-education.

We used a computerized block randomization procedure (allocation ratio 1:1, block size 10). The researcher conducting the randomization had no information about the participants apart from their 6-digit codes and did not participate in the enrollment and assignment of the participants to study groups, which was handled by two different researchers. Outcome variables were assessed at baseline (T0) and 12 weeks after randomization (post-treatment, T1). In addition, a follow-up measurement was assessed 24 weeks after randomization (12 weeks after treatment, T2). Sample size calculation was based on expected between-group differences at follow-up. G*Power was used for sample size calculation [34]. First, we assumed a power of 0.80, an alpha level of 0.05, and a small to medium effect size (*d*=0.3), which results in N=357 to perform a two-sided *t* test for differences between two independent means. Second, adding 20% attrition rate at inclusion, post-assessment, and follow-up, we calculated that N=608 participants needed to be enrolled.

The study was approved by the ethical review board at Leuphana University of Lüneburg. The study was registered retrospectively on February 1, 2013, under the International Standard Randomized Controlled Trial Number ISRCTN02446836; http://www.controlled-trials.com/ISRCTN02446836. Despite retrospective registration, no participants were enrolled before registration.

### Recruitment

Participants were recruited from a large-scale German statutory health insurance between January 2013 and April 2014, with the first participant enrolled in February 2013. We recruited members from Kaufmännische Krankenkasse (KKH), a statutory health insurance company with about 1.8 million members nationwide. First, to identify participants who were at high risk for sick leave due to depression, insurance members were screened for previous diagnosis of depression (International Classification of Disease codes F32.0, F32.1, F33.0, F33.1, and F34.1), previous sickness absence due to depression, and current sickness absence. Second, the study team sent an invitation letter to all positively screened insurance members along with study information, the informed consent form (see Multimedia Appendix 1), and a 6-digit code to login into the platform. Adults with a previous episode of mild to moderate depression (International Classification of Disease codes F32.0, F32.1, F33.0, F33.1) or dysthymia (F34.1) were included to avoid giving less intensive treatment than necessary. Before registration on the platform, a screening for exclusion criteria was performed. Participants with a score of ≥20 on the Patient Health Questionnaire (PHQ-9), indicating severe depression, were excluded. A second exclusion criterion was suicidality as measured by one item on the presence of suicidal thoughts. All participants had unrestricted access to treatment as usual during the study period, including access to the treatments and services which are typically available for depression in the German health care system (eg, psychotherapy and medication).

### Intervention

The Web-based intervention “HelpID” is a 12-week, Web-based program based on cognitive-behavioral therapy, awareness training, and systemic counseling. The program was structured into 12 weekly sessions. Each lasted 30 to 45 minutes and included interactive elements, videos, and audios that explained depression-related themes (eg, symptoms and course) as well as graphs, illustrations, exercises, and guidance for awareness and relaxation. Each session was available one week after completing the prior session. Participants received weekly reminder emails when a new session was available. The program had a guided format with therapist contact upon request, that is, psychologists (bachelor level or higher) trained in the intervention approach provided feedback via email or telephone. The intervention was developed by a team of clinical psychologists headed by Dr Despina Lion, a clinical psychologist and therapist with extensive experience in systemic counseling, cognitive-behavioral therapy, and neurological psychology. It is accessible online [35] (see Multimedia Appendix 2). Since July 2016, the copyright of “HelpID” is owned by IVPNetworks GmbH, a private integrated care company. The intervention is commercially available to single users and is included in health care plans of statutory health insurances.

The intervention’s psychological approach includes cognitive-behavioral therapy, mindfulness training, and systemic counseling. During the development process, current research evidence on the respective therapies was used as the basis, and special emphasis was placed on a “person-based” approach, focusing on the perspectives of the people who would use the intervention. Cognitive-behavioral therapy is the most extensively researched psychological treatment approach in Web-based interventions [36]. From cognitive-behavioral therapy, the intervention used elements of cognitive restructuring, with an emphasis on dealing with negative moods and automatic thoughts, as well as exercises for behavioral activation. Mindfulness training has been used increasingly in psychotherapy over the past years. It was shown to be effective for depressive symptoms and can be adapted to online formats [37,38]. The intervention module on mindfulness engages the user in exercises to observe the self and to practice mindfulness in daily situations. Systemic counseling is a therapeutic approach that highlights the social context surrounding the individual and its resources [39]. Specifically, systemic questioning techniques and instructions were employed to make use of the participants’ social support. Systemic principles were presented in specific weekly sessions, while homework exercises on systemic therapy encouraged the participants to adopt a systemic viewpoint and behavior change in their everyday interactions.

### Control Group

The control group was a wait-list plus psycho-education condition. During the 12-week study period, participants had access to text-based information on the nature of depression and its symptoms and treatment. The psycho-education content was developed by a team of trained psychologists (bachelor degree or higher) and was based upon scientific literature on depression (eg, the German S3-Guideline) [40]. This type of control condition was chosen because more active control groups (ie, psycho-education) are considered to be more methodologically valid than passive control groups (ie, wait-list conditions) [24]. There is evidence that psycho-education can reduce depressive symptoms and serve as an initial treatment in primary care [41]. The control group did not have access to therapist guidance. Participants were eligible to access the intervention after study completion, if they requested access.

### Outcomes

The primary outcomes were self-assessed depressive symptoms with the Patient Health Questionnaire (PHQ-9) and the Beck Depression Inventory (BDI II). The PHQ-9 measured the severity of depressive symptoms over the preceding 2 weeks, resulting in a score between 0 to 27 points with higher values indicating more severe depression [42,43]. The PHQ-9 was shown to have good reliability and construct validity [42]. The BDI II uses 21 items to measure depression severity [44,45]. The BDI II showed good psychometric properties in German-speaking samples in regard to internal consistency, retest-reliability, and construct validity [46]. As a secondary outcome, quality of life was assessed using the Manchester Short Assessment of Quality of Life (MANSA) [47]—a 12-item scale rating the participants’ satisfaction with different life domains. The MANSA has been validated in a Swedish sample and showed satisfactory internal consistency and construct validity [48]. User satisfaction was measured at post-assessment using the item “Overall, how satisfied are you with the program?” with four answer options: 1=very good, 2=good, 3=satisfactory, and 4=poor.

Information on work absenteeism was retrieved from health insurance records. In the German health care system, such standardized health data is collected routinely. Its primary purpose is cost reimbursement and quality assurance, but it can be made available for secondary analysis. Due to the routine data collection, health insurance records are assumed to have high ecological validity. We matched health insurance records from KKH health insurance with participants’ data using a 6-digit participant code as identifier. The code was generated for each positively screened insurance member and was also used for registration on the study platform. We analyzed sickness absence data that covered the 90 days before randomization (baseline) and 90 days after intervention (post-assessment). Three sickness absence measures were constructed according to Hensing et al [33]. First, the number of persons who were absent at least once, second, absence frequency as the number of times a person was absent during the 90 day period irrelevant of duration, and third, absence duration as the total number of absence days during the 90 day period. Sickness absence data was not diagnosis-specific.

### Statistical Analysis

Statistical analysis was performed on an intention-to-treat (ITT) basis according to the recommendations in the CONSORT statement [49] and its adaption for eHealth trials [50] (see Multimedia Appendix 3). Missing data at post-treatment was imputed using the Markov Chain Monte Carlo multiple imputation (missing data module in IBM SPSS version 22), where 10 estimations per missing value were specified and, besides the outcome variables, group assignment was included as an additional variable. Under the assumption that data is missing at random, multiple imputation was considered suitable to produce more precise estimates of the true intervention effect than other imputation methods, that is, last observation carried forward [51]. In addition, per-protocol (PP) analysis was performed to examine the robustness and sensitivity of the findings when including only participants who completed the post-assessment. *t* tests were used to determine differences in baseline characteristics and for within-group differences. The difference in the intervention outcomes between the intervention group and the control group at post-treatment was estimated using analysis of covariance (ANCOVA) with baseline scores as the covariate. Cohen *d* was calculated as a measure of the effect size, using pooled standard deviations [52]. For the between-group effect sizes, Cohen *d* was computed from the mean differences.

To assess clinical significance on an individual level, the reliable change index (RCI) was computed for the PHQ-9 [53]. Cronbach alpha=.89 from Kroenke et al [42] was used as an estimate of the reliability of the PHQ-9, along with pre-treatment standard deviations from the current study. Participants were classified as “responders” if they displayed a reliable positive change, or as “deteriorated” if they displayed a negative change on the RCI. A reliable positive change corresponds to less than −1.96 on the RCI and a change of PHQ-9 points to greater than −4.05. A reliable negative change corresponds to less than −1.96 on the RCI and a change in PHQ-9 points to greater than 4.05. Finally, the number needed to treat (NNT) [54] was computed.

All analysis was performed using Stata 13. The reported *P* values are two sided and in the 95% CI.

**Figure 1:**
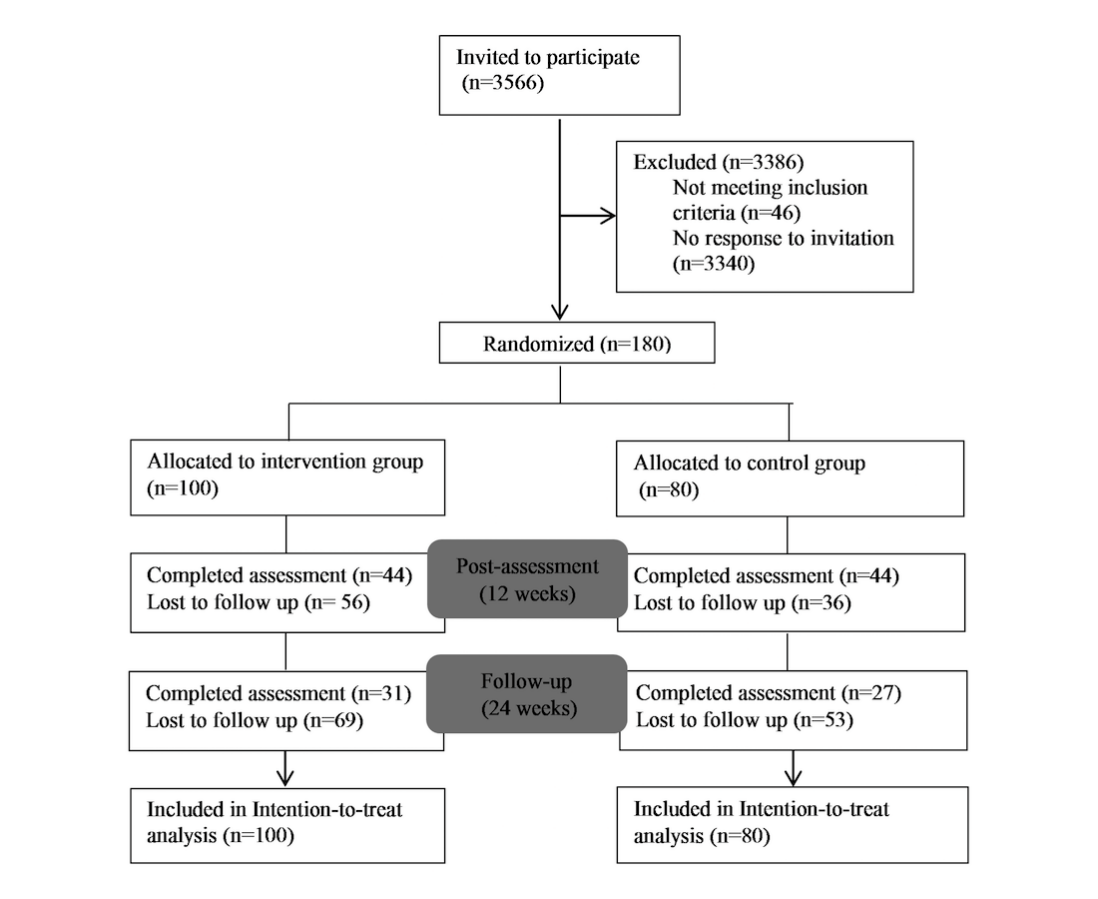
Study flowchart.

## Results

### Participant Characteristics

Figure 1 shows the flow of participants through the study. The complete pool of insurance members was screened, which resulted in 3929 positively screened insurance members who were subsequently invited to participate. Of those, 180 responded, met the inclusion criteria, provided informed consent, and were randomized. Of the 180 participants, 88 completed the post-assessment after 12 weeks (retention rate: 48.8%, 88/180), and 58 completed the follow-up assessment after 24 weeks (retention rate: 32.2%, 58/180). To estimate achieved power, a post-hoc power analysis was conducted. This revealed that with the sample of 180 participants, the achieved power to detect an effect of *d*=0.3 was 0.51.

Baseline characteristics between participants who completed the post-assessments and those who were lost to follow-up were tested for differences. Older participants (PHQ at T1: *P*=.02, BDI at T2: *P*=.03) and participants with higher education (PHQ at T1: *P*=.03, BDI at T2: *P*=.04) were more likely to complete the post-assessment on the primary outcome and the follow-up assessment. Participants who were not in psychotherapy during study enrollment were more likely to complete post-assessment on one of the primary outcomes (BDI at T1: *P*=.04) and the follow-up assessment (BDI at T2: *P*=.04) as compared with participants who were on a waiting list or in psychotherapy at enrollment. No other relevant differences between those who completed the post-assessments and those who were lost to follow-up were found.

Table 1 shows the participant characteristics at baseline. Participants had an average age of 48 years and were predominantly female (68%). The majority were married or had a partner (56%) and had completed secondary education or higher (85%). About half of the sample was working full-time (51%) and another 27% were working part time, while 20% of those working had an executive position. About one-fifth (21%) were not working. The majority had experienced a previous depressive episode (51%) or reported to have chronic depression (30%). During study enrollment, half of the participants were prescribed with depression medication (50%), 26% were in psychotherapy, and 13% were on a psychotherapy waiting list. Of the total number of enrolled participants, 43% had a sick leave certificate at the time. The mean baseline depressive symptoms in the sample were 11.10 points on the PHQ-9 (SD=4.45), indicating moderate depression. No clinically relevant differences in terms of any baseline characteristics were found, and we concluded that randomization was successful.

**Table 1:** Participant characteristics at baseline.

Characteristic		Intervention group	Control group	Total sample	*P* value
		n=100^a^	n=80	n=180	
PHQ-9 Score, mean (SD)		11.53 (4.35)	10.56 (4.53)	11.10 (4.45)	.15
Age, mean (SD)		47.01 (10.36)	48.66 (11.59)	47.74 (10.92)	.31
**Gender, n (%)**					
	Male	34 (34.0)	23 (28.7)	57 (31.7)	.45
	Female	66 (66.0)	57 (71.3)	123 (68.3)	
**Relationship, n (%)**					
	Single	24 (24.0)	18 (22.5)	42 (23.3)	.93
	Married/Partner	56 (56.0)	46 (57.5)	102 (56.7)	
	Divorced/Separated	15 (15.0)	14 (17.5)	29 (16.1)	
	Widowed	5 (5.0)	2 (2.5)	7 (3.9)	
**Education, n (%)**					
	Low	13 (13.3)	13 (16.2)	26 (14.6)	.38
	Middle	63 (64.3)	42 (52.5)	105 (59.0)	
	High	22 (22.4)	25 (31.3)	47 (26.4)	
**Employment, n (%)**					
	Full-time	51 (52.0)	39 (50.6)	90 (51.4)	.41
	Part-time	30 (30.6)	18 (23.4)	48 (27.4)	
	Not working	17 (17.3)	20 (26.0)	37 (21.2)	
**Executive position, n (%)**					
	Yes	20 (22.0)	13 (17.6)	33 (20.0)	.48
	No	71 (78.0)	61 (82.4)	132 (80.0)	
**Previous depression^b^, n (%)**					
	None	18 (18.0)	14 (17.7)	32 (17.9)	.53
	Episodic	54 (54.0)	38 (48.1)	92 (51.4)	
	Chronic	28 (28.0)	27 (34.2)	55 (30.7)	
**Depression medication, n (%)**					
	Yes	53 (53.0)	36 (45.6)	89 (49.7)	.33
	No	47 (47.0)	43 (54.4)	90 (50.3)	
**In psychotherapy, n (%)**					
	Yes	30 (30.0)	16 (20.3)	46 (25.7)	.15
	No	57 (57.0)	52 (65.8)	109 (60.9)	
	Waiting List	13 (13.0)	11 (13.9)	24 (13.4)	

^a^All values (except for *P* values) are mean (SD) or n (%).

^b^Original item: “Did you have these symptoms for the first time?” Answer options: 1. “Yes,” 2. “No; I had one or multiple episodes,” 3. “No; the symptoms last for several years.”

### Intervention Effectiveness

Table 2 shows the mean scores, standard deviations for the intervention outcomes at baseline and at post-assessment, effect sizes, and statistical significance, based on the intention-to-treat sample (imputed data). A significant between-group difference in favor of the intervention group was found for the PHQ-9 (*F*_1,179_=15.06, *P*<.001), which corresponds to a medium effect size (*d*=0.55, CI 0.25-0.85). For those in BDI-II, a significant between-group difference at post-treatment was found (*F*_1,179_=8.69, *P*=.004), which corresponds to a moderate effect size (*d*=0.40, CI 0.10-0.70).

**Table 2:** Means, standard deviations (SD), effect sizes, and statistical significance for intervention outcomes based on intention-to-treat sample (imputed data).

Outcome		Mean (SD)				Effect Size^a^		Statistical Significance^b^	
		Baseline (T0)	Post-assessment (T1)	Difference (T0−T1)	Cohen *d* (95% CI)		*F* Value		*P* value
**PHQ-9**									
	Intervention	11.53 (4.35)	6.51 (2.87)	−5.02 (3.62)	0.55 (0.25-0.85)		15.06		<.001
	Control	10.56 (4.53)	7.76 (3.63)	−2.80 (4.42)					
**BDI-II**									
	Intervention	20.07 (7.99)	13.55 (6.46)	−6.17 (6.39)	0.41 (0.11-0.70)		8.69		.004
	Control	18.78 (9.84)	15.52 (8.62)	−3.56 (6.68)					
**MANSA**									
	Intervention	3.27 (0.72)	3.50 (0.67)	0.14 (0.71)	0,12 (−0.17 to 0.42)		0.72		.39
	Control	3.30 (0.85)	3.44 (0.70)	0.22 (0.55)					

^a^Between-group effect size from mean differences.

^b^Based on ANCOVA controlling for baseline scores (T0).

In addition, both the intervention and the control group showed reductions in depressive symptoms as measured by within-group changes from baseline to post-assessment. In the intervention group, a mean reduction of 5 points on the PHQ-9 was found (*t*_99_=14.28, *P*<.001), which corresponds to a large within-group effect size (*d*=1.42, CI 1.14-1.71). In the control group, a mean reduction of 2.79 points was found (*t*_79_=5.82, *P*<.001), corresponding to a moderate effect size (*d*=0.65, CI 0.41-.89).

In the per-protocol analysis, we tested for differences in PHQ-9 scores between intervention completers and noncompleters at post-assessment. No significant difference was found (*t*_178_=−.28, *P*=.78). Table 3 presents the results of the per-protocol analysis. For the PHQ-9, a significant between-group difference in favor of the intervention group was found among completers (PHQ-9: *F*_1,77_=8.98, *P*=.04), corresponding to a moderate effect size (*d*=0.61, CI 0.15-1.07). The mean PHQ-9 scores among completers were reduced by 5.70 points in the intervention group and by 2.24 points in the control group—this corresponds to a large effect size (*d*=1.72, CI 1.23-2.22) and a moderate effect size (*d*=0.49, CI 0.14-0.82) for within-group changes, respectively. For the BDI-II, the between-group effect failed to reach statistical significance in the per-protocol analysis (*F*_1,77_=0.81, *P*=.37, *d*=0.25, CI −0.18 to 0.65). BDI-II within-group changes were significant in the intervention group (*t*_43_=3.68, *P*<.001) and the control group (*t*_42_=4.70, *P*<.001).

**Table 3:** Means, standard deviations (SD), effect sizes, and statistical significance for intervention outcomes based on per-protocol sample (nonimputed data).

Outcome		Mean (SD)				Effect Size^a^		Statistical Significance^b^	
		Baseline (T0)	Post-assessment (T1)	Difference (T0−T1)	Cohen *d* (95% CI)		*F* value		*P* value
**PHQ-9**									
	Intervention	11.53 (4.35)	6.50 (3.85)	−5.03 (3.29)	0.61 (0.15-1.07)		8.98		.04
	Control	10.56 (4.53)	7.95 (4.62)	−2.61 (4.61)					
**BDI-II**									
	Intervention	20.07 (7.99)	14.86 (8.05)	−5.21 (7.59)	0.25 (−0.18 to 0.65)		0.81		.37
	Control	18.78 (9.84)	15.32 (10.34)	−3.46 (6.32)					
**MANSA**									
	Intervention	3.27 (0.72)	3.52 (0.86)	0.25 (0.64)	0.18 (−0.22 to 0.58)		0.34		.56
	Control	3.30 (0.85)	3.42 (0.83)	0.12 (0.79)					

^a^Between-group effect size from mean differences.

^b^Based on ANCOVA controlling for baseline scores (T0).

**Table 4:** Work absence indicators at baseline and at post-assessment for 160 participants.

Indicator		Baseline (T0)^a^	Post-assessment (T1)	Difference (%)	*P* value
**Absence at least once, n (%)**					
	Intervention	75 (85.22)	25 (28.41)	−50 (−66.67)	<.001
	Control	58 (80.55)	12 (16.67)	−46 (−79.31)	<.001
**Absence frequency, mean (SD)**					
	Intervention	1.19 (0.09)	0.39 (0.08)	−0.80 (−67.23)	<.001
	Control	1.15 (0.10)	0.20 (0.06)	−0.95 (−82.61)	<.001
**Absence duration, mean (SD)**					
	Intervention	25.60 (2.03)	24.65 (3.80)	−0.95 (−3.71)	.79
	Control	27.69 (2.37)	24.04 (4.36)	−3.65 (−13.18)	.34

^a^Baseline and post-assessment cover a period of 90 days each.

### Treatment Response

In the intervention group, 63% (63/100) of the participants showed a reliable symptom change from baseline to post-intervention and were thus classified as responders. In the control group, 33% (27/80) were classified as responders. The difference in reliable symptom change between intervention and control group was significant (*t*_178_=3.39, *P*<.001). This resulted in a NNT of 4.08. One participant in the intervention group experienced symptom deterioration, and five participants in the control group experienced symptom deterioration.

### Sickness Absence

Information on sickness absenteeism was available for 160 participants (Intervention group: n=88, control group: n=72). For 20 participants, sickness absenteeism could not be retrieved from insurance records, and therefore the data from these participants was unavailable.

Table 4 shows persons who were absent at least once, with absence frequency and absence duration at baseline and at post-assessment. The majority of the participants were absent at least once during the baseline period: IV: 85% (77/85), CG: 80% (58/72). Overall, significantly fewer participants were absent at least once during post-assessment (IV: 28%, CG: 16%). The within-group absence reductions were significant (IV: *t*_87_=6.54, *P*<.001, CG: *t*_71_=6.17, *P*<.001).

Regarding absence frequency, participants in the intervention group were absent on average 1.2 times at baseline and 0.4 times at post-assessment. In the control group, participants were absent 1.2 times at baseline and 0.2 times at post-assessment. The within-group reductions in absence frequency were significant (IV: *t*_87_=7.49, *P*<.001, CG: *t*_71_=8.59, *P*<.001).

Similarly, high absence durations at both baseline and post-assessment were found. From the 90 days examined at each time point, participants in the intervention group were absent from work 26 days at baseline and 25 days at post-assessment. In the control group, participants were absent 28 days at baseline and 24 days at post-assessment. However, there were no significant differences in absence duration from baseline to post-assessment (IV: *t*_87_=.26, *P*=.79, CG: *t*_71_=.95, *P*=.34). For all three measurements, the between-group differences at post-assessment failed to reach statistical significance (absence at least once: *F*_1,159_=.80, *P*=.37, absence frequency: *F*_1,159_=3.24, *P*=.07, absence duration: *F*_1,159_=.02, *P*=.88).

### Secondary Outcome

No significant difference in quality of life as measured by MANSA was found (*F*_1,169_=.71, *P*=.40, *d*=0.13, CI −0.21 to 0.41).

### Long-term effect

No significant between-group difference for BDI-II depression scores at 24-week follow-up was found (*F*_1,85_=.81, *P*=.33). However, significant within-group changes were sustained for both the intervention and the control group. In the intervention group, there was a mean reduction from baseline to follow-up assessment of 5.46 points on the BDI-II (*t*_99_=6.81, *P*<.001), which corresponds to a moderate effect size (*d*=0.68, CI 0.46-0.90). In the control group, there was a mean reduction of 4.69 points (*t*_79_=4.37, *P*<.001), corresponding to a small effect size (*d*=0.48, CI 0.26-0.72).

### User Satisfaction

87 participants completed the user satisfaction survey at post-assessment. In the intervention group, 13.6% (6/44) rated the program overall as very good, 68.2% (30/44) as good, and 18.2% (8/44) as satisfactory. In the control group, 4.6% (2/43) rated the program as very good, 37.2% (16/43) as good, 34.9% as satisfactory, and 23.3% (10/43) as poor. Mean satisfaction scores were 2.04 in the intervention group and 2.76 in control group. There was a significantly higher mean satisfaction in the intervention group (*t*_85_=4.60, *P*<.001).

## Discussion

### Principal Findings

This study compared the effectiveness of a Web-based intervention in reducing depressive symptoms and sickness absence among adults with immediate risk for sickness absence due to mild to moderate depression. When compared with a wait-list plus psycho-education control group, participants who used the Web-based intervention showed a significantly greater reduction in depressive symptoms. However, because of the low response and high attrition in this study, one primary outcome did not reach statistical significance among participants who completed the intervention (per-protocol analysis) and at follow-up, only within-group changes were sustained but the intervention effect was not. In terms of individual clinical significance, significantly more participants in the intervention group responded to the treatment. We used health insurance records to measure sickness absence and found that sickness absenteeism declined in both groups, but there were no statistical differences in work absence between groups. The achieved power of this trial was lower than calculated. Therefore, its results should be replicated in a larger sample.

### Comparison With Prior Work

These findings are especially relevant when considering the increasing impact of mental ill-health on workforces across industrialized countries [55]. Depression is a significant cause for workday losses and produces more absence durations than other mental illnesses. To reduce the illness burden, widespread access to evidence-based treatment is needed to maintain workers’ mental health before companies and individuals face more serious burdens as the illness progresses. In general, Web-based interventions provide a promising treatment tool because these interventions can be accessed at any time and at different locations at the users’ own pace. Due to their relative anonymity, Web-based interventions may especially benefit employees with depression who wish to avoid the negative perceptions of being mentally ill in the workplace. However, risks associated with the dissemination of Web-based interventions in the health care system have been reported as well. According to meta-analysis, the effects of Web-based interventions vary, making it necessary to evaluate each intervention separately. If Web-based interventions that are effective in reducing depressive symptoms are more widely implemented and adopted, a positive impact on the burden and impairment caused by depression can be expected. It can also help to overcome the shortcomings of conventional treatment (eg, waiting lists).

This study contributes to the growing body of research that supports the effectiveness of Web-based interventions for depression. Within this research, a critical mass of efficacy studies is needed to identify subgroups for which these interventions work [56]. Adults who are at high risk for sick leave from work due to mild to moderate depression have not yet been targeted specifically by Web-based interventions. In terms of effect sizes, previous research has found significant heterogeneity between studies. This makes it necessary to evaluate each intervention separately. The effect sizes reported in this study are comparable to other studies evaluating guided interventions for depression, including those included in the meta-analysis by Andersson and Cuijpers [23] where a mean between-group effect size of *d*=0.41 is reported. The amount of therapy guidance that is necessary to increase intervention effectiveness and adherence remains a subject of debate in the field of Web-based interventions [18,57]. Considering that this intervention provided only minimal therapist support upon request and achieved similar outcomes to studies with more intense guidance, we speculate that merely having the option to contact a therapist during the intervention—versus regular therapist contact—is sufficient for the needs of many participants and works equally well. However, it is possible that the number of dropouts could have been reduced with regular guidance. The examination of support preferences was not within the scope of this study and further research on this subject is needed.

We found that health insurance records are a suitable outcome for effectiveness research in Web-based interventions. Both groups showed reductions in work absence frequency, however, no statistical difference in work absence between groups was found. Several explanations may account for this finding. First, we cannot rule out the possibility that the decline in work absence frequency over time was caused by regression toward the mean or spontaneous remission. Our sample was recruited during a period of high levels of sickness absence, as seen in the data (see Table 3). Consequently, due to statistical chance, the frequency of sickness absence tended to approach lower levels at post-assessment. A healthy control group is needed to compare baseline levels of sickness absence, which was unavailable in this study. Second, it is possible that the 90-day time period in our study was not sufficiently long to appropriately detect changes in work absence. Previous population studies on sickness absence due to mental health problems found a median absence duration of 79 days [58]. This indicates that sickness absence started or ended outside of the time period of this study. Similarly, our sample was not adequately powered to detect small differences in work loss days. Third, organizational factors (ie, high work demands, job security) could have influenced work absence in this study. Unfortunately, we could not measure organizational variables.

To disentangle these explanations, future studies on the effect of Web-based interventions on work absence should include a longer time period, information on organizational factors that may be related to sickness absence, and work absence data from a healthy control group for baseline comparisons. Integrating objective behavioral parameters (ie, sickness absence data from health insurance companies) can increase the validity of effectiveness studies and might be a valuable addition to self-reported outcome measurements.

### Privacy and Data Security

In Web-based interventions, health-related information is processed and stored electronically. Therefore, data security and confidentiality issues need to be taken seriously. This study used several measures to ensure the privacy of the study participants. Person-related information and study data were stored on separate servers to ensure that individuals could not be identified. Communication between the users’ Web browsers and the servers were encrypted via a Secure Sockets Layer (SSL) connection. All data were stored on servers located in Germany.

### Limitations

There are several limitations to this study that must be acknowledged. First, although reporting the effect of the Web-based intervention was within the scope of our study, it remains unclear as to which specific elements and properties of the intervention contributed to its effectiveness. Regarding the length of the intervention, evidence on the dose-response relationship in psychotherapy points to the conclusion that most progress occurs in the first few sessions of an intervention [59]. Similarly, Web-based interventions with 8 or less sessions were found to be more effective than interventions with more than 8 sessions [21]. This indicates that the present intervention with its 12 sessions could be shortened in length while maintaining its effectiveness.

A second limitation concerns the response rate. Response from the pool of positively screened insurance members was low (5.8%, 226/3929). This raises the concern that participants were particularly motivated to use a Web-based intervention when compared with nonparticipants, especially because nonparticipants were positively screened for sickness absence due to depression and thus belonged to the target group. Ideally, data from nonparticipants should have been collected as a baseline comparison group, but this data was unavailable in this study because of a lack of informed consent. When the complete pool of insurance members was screened and all positively screened insurance members were invited, recruitment was stopped. This resulted in a substantially smaller sample than was previously calculated in the power analysis (calculated N=680 vs actual N=180).

Third, attrition during the study was high. At post-assessment, 45.5% of the participants had dropped out, and at follow-up, 67.7% of participants had dropped out. In general, dropout is a common problem in Web-based interventions [60]. However, the dropout in this study was remarkably higher than the average attrition rate for Web-based interventions with therapist support, as reported in the meta-analysis by Richards and Richardson (28%) [21]. Several recent studies on Web-based interventions showed dropout rates that were remarkably lower [27,32]. One possibility for the relatively high attrition in this study is that participants who failed to complete a weekly session were reminded via email only. Comparable studies, which used telephone reminders, achieved substantially higher participant compliance. A second explanation is that therapist guidance was available upon request only. Guided interventions have lower attrition rates as compared to unguided interventions. Thus, it is possible that participants who were at risk for dropout were less likely to use therapist guidance. Despite our analysis of per protocol and imputed data showing comparable results, which indicates no difference in intervention completers versus noncompleters, the risk remains that study dropout could have biased the results. Due to the associations of several baseline characteristics (age, education, and being in psychotherapy) with the likelihood to complete the outcome assessments, the missing at random assumption could have been violated. Overall, the high attrition rates limit the conclusions drawn from this study.

Third, the positive relationship of age and education with study dropout seen here limits the generalizability of the findings to younger and less educated groups. This is supported by the composition of the study sample, where highly educated participants and those in executive positions are over-represented. Further studies with more statistical power are needed to identify effectiveness among different subgroups.

Fourth, participants had access to treatment as usual during the study, including psychotherapy and medication. Therefore we cannot exclude the possibility that within-group changes in depression scores were affected by third factor variables. Thus, within-group changes must be interpreted with caution.

Fifth, no clinical interviews were conducted to assess depression. Structured clinical interviews represent the gold standard of clinical assessment, with superior validity and reliability. Due to limited resources, this study relied solely on participants’ self-reports to assess clinical symptoms.

Sixth, the amount and duration of provided therapeutic support during the study was not measured, making it difficult to compare the results with other studies on Web-based interventions that have used different levels of support, ranging from no support to more intensive and regular support. In this study, support was provided upon request, which could have prevented some participants from using support, thus lowering adherence.

Seventh, we used a wait-list plus psycho-education control group. Wait-list control groups undermine internal validity and may lead to an over-estimation of the treatment effect [24]. Thus, active control groups are considered to be less biased. To maximize participant response, we decided to inform control group participants that they could access the intervention upon request after study completion. This may have lowered expectations with regard to the control condition. Furthermore, control group participants were active during the study period as they had access to psycho-education. As a result, we observed mean symptom reductions in the control group, which is consistent with the finding that Web-based psycho-education can reduce depressive symptoms [41]. At the same time, it is possible that psycho-education has adverse effects in some participants, because it sensitizes patients to the topic of depression, leading to an over-reporting of symptom severity at follow-up. For example, a study that used a psycho-education control group found that the incidence of depression was higher than usual [32]. Our results show that reliable symptom deterioration was low overall, but occurred more frequently in the control group (6.25%, 5/80) as compared with the intervention group (1%, 1/100). This suggests that adverse effects in the control groups were present, but were unlikely to bias the treatment effect. As we did not collect data on usage and engagement with the psycho-education in the control condition, the perceived credibility of the psycho-education remains unknown.

Eighth, this was an open-label trial, where participants and researchers were aware of which group was receiving which treatment. Furthermore, only questionnaire data was assessed as a proxy of use parameters, but no uptake data was available on the actual usage of the program (eg, frequency and length of website usage). Data on how the participants interacted with the program could provide valuable insights into the effectiveness of specific intervention elements.

### Conclusions

The Web-based intervention reduced depressive symptoms among adults with sickness absence. As this trial achieved a lower power than calculated, its results should be replicated in a larger sample. Further validation of health insurance records as an outcome measure for eHealth trials is needed.

## Appendix

Multimedia Appendix 1 Patient information informed consent form.

Multimedia Appendix 2 Intervention screenshots.

Multimedia Appendix 3 CONSORT-EHEALTH-checklist.

## Abbreviations

ANCOVA: analysis of covariance
BDI-II: Beck Depression Inventory
ITT: intention-to-treat
KKH: Kaufmännische Krankenkasse
MANSA: Manchester Short Assessment of Quality of Life
PHQ-9: Patient Health Questionnaire
PP: per-protocol
RCI: Reliable Change Index
