# Supplementary material for: Effectiveness of a Web-Based Intervention in Reducing Depression and Sickness Absence: Randomized Controlled Trial
Source: J Med Internet Res. 2017 Jun 15;19(6):e213. doi: 10.2196/jmir.6546 (PMC5491897; doi:10.2196/jmir.6546)
Supplement: Multimedia Appendix 1 [file jmir_v19i6e213_app1.pdf]

„Unterstützung aus dem Internet: Eine Studie zu Onlineangeboten für Menschen in seelischen Belastungssituationen“

- Informationsblatt -

Sehr geehrte Damen und Herren,

immer häufiger sind Menschen von einer Depression betroffen. In den meisten Fällen handelt es sich um ein vorübergehendes Problem, das nach kurzer Zeit wieder verschwindet. Manchmal ist der Leidensdruck jedoch sehr stark und kann zu Beeinträchtigungen im Alltags- oder Berufsleben führen. Gesundheitsbezogene Informationen und Angebote im Internet gewinnen zunehmend an Bedeutung. Internetgestützte Programme zur Bewältigung von psychischen Erkrankungen haben viel Potential, sind jedoch hinsichtlich ihrer Wirksamkeit noch wenig erforscht. Die KKH unterstützt in diesem Zusammenhang eine Studie der Leuphana Universität Lüneburg, welche den Nutzen internetbasierter Unterstützungsangebote für Menschen mit Depressionen oder Belastungsreaktionen untersucht. Die Studie wurde vom Ethikbeirat der Leuphana Universität Lüneburg genehmigt.

Wir möchten Sie hiermit herzlich dazu einladen, an unserer Studie mitzuwirken.

Im Folgenden möchten wir Sie über den Hintergrund und die Ziele sowie den Nutzen und mögliche Risiken bei einer Teilnahme an der Studie informieren.

Worum geht es bei unserer Untersuchung?

Internetbasierte Depressionsprogramme können es Betroffenen erleichtern, für ihre psychische Gesundheit aktiv zu werden. Im Ausland werden derartige Onlineangebote bereits verstärkt eingesetzt. Sie beinhalten Informationen über die Krankheit Depression oder bieten Entspannungsübungen sowie Hinweise zu einer erfolgreichen Bewältigung der Erkrankung an.
Das Ziel unserer Studie ist, den Nutzen von Unterstützungsangeboten aus dem Internet für Menschen mit leichter bis mittelschwerer Depression oder Belastungsreaktion zu untersuchen. Hierzu vergleichen wir das neu entwickelte Selbsthilfeprogramm „HelpID“ der Novego AG und ein online zur Verfügung gestelltes Informationsangebot zum Thema Gesundheit und Depression.

Was ist „HelpID“?

„HelpID“ wurde mit dem Ziel entwickelt, Menschen mit einer Depression dabei zu unterstützen, ihren Gesundheitszustand zu verbessern. Die Arbeit mit „HelpID“, mit auf die individuellen Bedürfnisse des Nutzers abgestimmten wöchentlichen Sitzungen und Übungen, dauert 12 Wochen. Es werden Informationen zu spezifischen Themen rund um die Depression, Hinweise zur Bewältigung der Erkrankung sowie Achtsamkeits- und Entspannungsübungen angeboten. Zudem erhalten die Studienteilnehmer[1] auf Wunsch ein schriftliches Feedback zu den Übungen durch ein psychologisch geschultes Team.

[1] Aus Gründen der besseren Lesbarkeit wird im Text nur die männliche Form verwendet. Gemeint ist stets sowohl die weibliche als auch die männliche Form.

Europa fördert Niedersachsen
EUROPÄISCHE UNION
Europäischer Fonds für regionale Entwicklung

Was beinhaltet das „Online-Informationsangebot zum Thema Gesundheit und Depression“?

Das Angebot umfasst Informationen zum Thema Depression und zur Gesundheitsförderung. Über einen Zeitraum von 12 Wochen erhalten Sie einmal wöchentlich per E-Mail einen Link zu informativen Texten, die darüber aufklären, was Depression ist und was Ihnen helfen kann die Depression zu lindern. Sie erhalten Hinweise für eine gesunde Lebensführung und Anregungen, wie Sie besser mit einer depressiven Phase zurechtkommen können. Die Materialien werden Ihnen online über ein Studienportal zur Verfügung gestellt.

Was sollten Sie noch über die Studienteilnahme wissen?

Um die beiden Online-Unterstützungsangebote vergleichen zu können, wird eine so genannte „randomisierte kontrollierte“ Studie durchgeführt. Über ein Losverfahren (ähnlich einem Münzwurf mit einer Wahrscheinlichkeit von jeweils 50 % für „Kopf“ oder „Zahl“) wird dabei entschieden, welches Unterstützungsangebot Sie erhalten. Die Studienteilnahme erfolgt in beiden Fällen ausschließlich über das Internet und ist für Sie kostenlos. Wenn Sie dem reinen Informationsangebot zugelost werden, erhalten Sie nach Abschluss der Studie per E-Mail einen kostenlosen Zugang für „HelpID“.

Die Online-Unterstützungsangebote sind nicht gleichzusetzen mit einer ärztlichen bzw. therapeutischen Behandlung, sie sollen für Sie vielmehr eine zusätzliche Hilfestellung zu Ihrer regulären allgemeinmedizinischen Versorgung darstellen. Eine Therapie kann und soll durch sie nicht ersetzt werden. **Bitte nehmen Sie unbedingt auch während Ihrer Studienteilnahme weiter andere medizinische und therapeutische Leistungen (z. B. Medikamente oder Psychotherapie) und/oder Leistungen Ihrer KKH in Anspruch**.

Ergänzend empfehlen wir Ihnen, Ihren behandelnden Arzt über Ihre Studienteilnahme zu informieren. Hierfür haben wir Ihnen ein *gelbes Informationsblatt* beigelegt, das Sie Ihrem Arzt bei Ihrem nächsten Besuch mitbringen können. Für allgemeine Rückfragen und weitere Informationen zur Studie kann uns Ihr Arzt mit Ihrem Einverständnis gerne kontaktieren.

Bisher deutet nichts darauf hin, dass sich die Nutzung der Online-Unterstützungsangebote negativ auf Ihr Befinden auswirken könnte. Da wir dies im Einzelfall jedoch nicht ausschließen können, kontaktieren Sie uns bitte bei Beschwerden, die in Zusammenhang mit der Nutzung der Online-Unterstützungsangebote auftreten (**studie.depressionshelfer@leuphana.de**).
Bitte beachten Sie, dass eine **Studienteilnahme bei schweren depressiven Symptomen und/oder Suizidalität nicht möglich** ist, da die Online-Unterstützungsangebote hier **keine ausreichende Hilfe** bieten. Bitte wenden Sie sich in diesem Fall an:

- eine Person Ihres Vertrauens aus Ihrem näheren Umfeld oder einen Angehörigen
- Ihren Hausarzt, einen Psychotherapeuten oder Psychiater
- den Sozialpsychiatrischen Dienst in Ihrem oder dem nächstgelegenen Ort
- die Notaufnahme des nächstgelegenen Krankenhauses.

Sie können außerhalb der ärztlichen Sprechzeiten auch die Arztrufzentrale des ärztlichen Bereitschaftsdienstes/Notfalldienstes unter der kostenlosen Telefonnummer **116117** anrufen. Falls Sie sofortige Hilfe benötigen zögern Sie bitte nicht: unter der Telefonnummer **112** erreichen Sie die zentrale Rettungsleitstelle. Deutschlandweite Krisennotdienste sind rund um die Uhr und kostenfrei unter **0800 - 111 0 111** oder unter **0800 - 111 0 222** telefonisch zu erreichen. Im Internet finden Sie weiterführende Informationen auf www.telefonseelsorge.de oder auf www.kompetenznetz-depression.de/hilfe/erste_hilfe.htm.

Europa fördert Niedersachsen
EUROPÄISCHE UNION
Europäischer Fonds für regionale Entwicklung

Sie entscheiden **freiwillig und auf eigene Verantwortung**, ob Sie an unserer Studie teilnehmen möchten. Eine Patientenversicherung besteht nicht. Wenn Sie sich dagegen entscheiden, entstehen Ihnen daraus keinerlei Nachteile. Die Studienteilnahme setzt Ihre schriftliche Zustimmung voraus. Diese ist nur wirksam, wenn Sie selbst einwilligungsberechtigt sind, d. h. sich nicht in rechtlicher Betreuung befinden oder einem Bevollmächtigten unterstehen.

Um an der Studie teilnehmen zu können, benötigen Sie einen Zugang zum Internet und eine E-Mail-Adresse. Kosten, die Ihnen aus der Nutzung des Internets entstehen, können nicht erstattet bzw. übernommen werden. Sie können zu jedem Zeitpunkt ohne Angabe von Gründen Ihre Teilnahme an der Studie beenden. Hierfür benötigen wir als Angabe lediglich Ihren Studien-Code oder Ihre bei der Studienanmeldung angegebene E-Mail-Adresse. Eine einfache schriftliche Mitteilung an das Studienzentrum (postalisch oder per E-Mail an **studie.depressionshelfer@leuphana.de**) ist ausreichend.

Wie ist der Ablauf der Studie?

Sie erhalten über einen Studien-Code einen 12-wöchigen Zugang zu einem der beiden beschriebenen Online-Unterstützungsangebote. In wöchentlichem Rhythmus nehmen Sie entweder an den Sitzungen und Übungen von „HelpID“ teil oder bekommen über das Studienportal online die informativen Ratgebertexte zur Verfügung gestellt. Sie erhalten hierzu jede Woche eine Erinnerungsmail. Uns interessiert, ob Ihnen das Online-Unterstützungsangebot bei Ihren Beschwerden geholfen hat. Daher möchten wir Sie bitten zu Beginn, sechs Wochen nach Beginn und nach Ablauf der 12 Wochen einen Online-Fragebogen zu Ihrem psychischen und körperlichen Befinden auszufüllen. Um zu erfassen wie sich Ihr Befinden in den darauffolgenden Monaten verändert, möchten wir Sie auch drei und sechs Monate nach Beendigung Ihrer Nutzung eines der beiden Online-Unterstützungsangebote erneut online befragen. Das Beantworten der Fragebögen wird jeweils etwa eine halbe Stunde Zeit in Anspruch nehmen. Ihre Auskünfte sind für uns von größter Wichtigkeit, auch in Bezug auf Ihre Zufriedenheit mit den angebotenen Leistungen.

Für erweiterte statistische Auswertungen werden Ihre Studiendaten mit den Daten der KKH zu den von Ihnen in den sechs Monaten vor und nach der Teilnahme an einem der Online-Unterstützungsangebote in Anspruch genommenen Leistungen der gesetzlichen Krankversicherung (GKV) verknüpft. Die Daten werden den beteiligten Wissenschaftlern in pseudonymisierter Form und ausschließlich zu Auswertungszwecken zur Verfügung gestellt. Hierfür ist Ihre schriftliche Zustimmung auf der Einverständniserklärung erforderlich.

Datenschutz

Alle von Ihnen erhobenen Daten sind nur den beteiligten Wissenschaftlern zugänglich und unterliegen der Schweigepflicht. Für die Anmeldung zur Studie und zur Zusendung der wöchentlichen Erinnerungsmails ist die Angabe einer E-Mail-Adresse erforderlich. Diese sollte nach Möglichkeit keine Rückschlüsse auf Ihre Person zulassen. Ihre E-Mail-Adresse und der Abschnitt der Einverständniserklärung mit Ihrer Unterschrift werden getrennt von den Fragebogen-Daten gespeichert bzw. aufbewahrt, eine Weitergabe Ihrer persönlichen Daten an Dritte ist ausgeschlossen. Ihre Daten werden durch Zuordnung eines Studien-Codes in pseudonymisierter Form (d. h. ohne Namensnennung) in einer passwortgeschützten Datenbank auf einem gesicherten Server gespeichert. Die Auswertung der Daten erfolgt mit Hilfe eines Computerprogramms und ausschließlich zu Studienzwecken. Die Ihre Behandlung betreffenden durch die KKH dokumentierten Daten werden zur Verknüpfung mit den Studiendaten ebenfalls über den Studien-Code pseudonymisiert. Nach Auswertung der Daten liegen die Studienergebnisse dann in anonymisierter Form vor (d. h. ohne die Möglichkeit der Datenzuordnung zum Studien-Code).

Europa fördert Niedersachsen
EUROPÄISCHE UNION
Europäischer Fonds für regionale Entwicklung

LEUPHANA
Inkubator

Bei einem Studienrücktritt werden Ihre Daten vollständig gelöscht. Bei regulärer Beendigung Ihrer Studienteilnahme wird Ihre E-Mail-Adresse ca. ein Jahr nach der letzten Online-Befragung aus der Studiendatenbank gelöscht und der entsprechende Abschnitt der Einverständniserklärung vernichtet.

Wenn Sie an unserer Studie teilnehmen möchten, füllen Sie bitte die beiliegende Einverständniserklärung vollständig aus und schicken diese an uns zurück. Eine Kopie ist für Ihre Unterlagen bestimmt. Einen Rücksendeumschlag („Entgelt zahlt Empfänger“) haben wir für Sie beigefügt. Sie können den Umschlag mit der ausgefüllten Einverständniserklärung einfach in den nächsten Briefkasten einwerfen, eine Briefmarke ist nicht erforderlich.

Für Ihre Online-Anmeldung zur Studienteilnahme benötigen Sie einen für Sie gut verfügbaren Internetzugang und Ihren **Studien-Code**. Diesen finden Sie oben rechts auf der Einverständniserklärung. Sobald uns Ihre vollständig ausgefüllte Einverständniserklärung vorliegt schalten wir Ihren Studiencode frei und Sie erhalten von uns eine E-Mail zur Bestätigung. Eine ausführliche Anleitung für den Anmeldevorgang im Online-Studienportal finden Sie im beiliegenden **Flyer** zur Studie.

Haben Sie noch Fragen? Rufen Sie uns gerne an unter 04131.677-7887 (Mo. & Do. von 10 – 12:00 Uhr, Mi. von 16 – 18:00 Uhr) oder senden Sie eine E-Mail an studie.depressionshelfer@leuphana.de.

Wir danken Ihnen schon heute sehr herzlich für Ihre Mithilfe!

Mit freundlichen Grüßen

Prof. Dr. Wulf Rössler (Projektleiter)

Dr. Jörn Moock (Projektkoordinator)

Ihr persönlicher Studien-Code:
A12345

Einverständniserklärung

zur Teilnahme an einer wissenschaftlichen Studie

„Unterstützung aus dem Internet: Eine Studie zu Onlineangeboten für Menschen in seelischen Belastungssituationen“

Ich bin mit dem schriftlichen Informationsblatt über die Vorgehensweise der Studie informiert worden.

Teilnahmeerklärung

- Ich entscheide mich **freiwillig und auf eigene Verantwortung** für die Teilnahme an der Studie. Mir ist bekannt, dass keine Patientenversicherung besteht.
- Ich weiß, dass ich meine Zustimmung jederzeit ohne Begründung und ohne Nachteile für mich unter Angabe meines Studien-Codes oder meiner angegebenen E-Mail-Adresse schriftlich widerrufen kann.
- Mir ist bewusst, dass die Studienteilnahme keinen Therapieersatz darstellt und ich weiter medizinische und therapeutische Leistungen in Anspruch nehmen kann.
- Mir wurde empfohlen, meinen Arzt[1] mit dem beiliegenden „Informationsblatt für Ärztinnen und Ärzte“ über meine Studienteilnahme zu informieren und bei einer Zustandsverschlechterung zu kontaktieren.
- Ich weiß, dass per Los entschieden wird, ob ich an „HelpID“ teilnehme oder das Onlineangebot mit Informationstexten zu Gesundheit und Depression erhalte. In letzterem Fall bekomme ich nach Studienende per E-Mail einen Zugang für die Nutzung von „HelpID“.
- Ich bin einwilligungsfähig und habe keinen rechtlicher Betreuer (§ 1896 BGB) oder Bevollmächtigten.

Einwilligungserklärung

- Ich bin einverstanden per Online-Fragebogen Angaben zu meinem Befinden und meiner Zufriedenheit mit den Online-Unterstützungsangeboten zu machen. Die Befragungen werden vor Beginn, sechs Wochen nach Beginn sowie nach Beendigung der Nutzungsdauer und erneut drei und sechs Monate später durchgeführt. Hierzu wird mich das Studienzentrum per E-Mail anschreiben.
- Ich bin einverstanden, dass die von mir erhobenen Daten **pseudonymisiert** werden (durch Zuordnung eines Codes und ohne Nennung meines Namens oder anderer personenbezogener Angaben) und damit keine Rückschlüsse auf meine Person zulassen. Meine Daten werden durch die beteiligten Wissenschaftler ausschließlich zu Studienzwecken gespeichert und ausgewertet. Meine persönlichen Angaben (E-Mail-Adresse und Unterschrift) werden vertraulich behandelt. Ich weiß, dass eine Weitergabe meiner Daten an Dritte ausgeschlossen ist.
- Ich stimme zu, dass die Leuphana Universität Lüneburg die KKH über meine Einverständniserklärung informiert und willige ein, dass die KKH Versicherungsdaten, die meine in Anspruch genommenen medizinischen Leistungen sechs Monate vor und nach meiner Studienteilnahme betreffen, pseudonymisiert an die Leuphana Universität Lüneburg weitergibt, damit diese mit den von mir im Rahmen der Studie erhobenen Daten verknüpft und in die Auswertung einbezogen werden können.

Die gesamte Untersuchung unterliegt den rechtlichen Bestimmungen zum Datenschutz.

Bitte senden Sie das unterschriebene Formular im beiliegenden Rücksendeumschlag an:

Innovations-Inkubator/KT „Vernetzte Versorgung“: Zusatzmodul „Onlinetherapie“
Leuphana Universität Lüneburg
Stichwort: „Online-Studie“
Rotenbleicher Weg 67
21335 Lüneburg

Durch meine Unterschrift erkläre ich mich einverstanden, an der Studie teilzunehmen und bestätige meine Einwilligung in die beschriebene Datenerhebung, -verarbeitung und -nutzung im Rahmen der Studie.

Ort, Datum, Unterschrift:

..

Wichtig! Bitte tragen Sie hier Ihre (möglichst anonyme) E-Mail-Adresse ein:

..

[1] Aus Gründen der besseren Lesbarkeit wird im Text nur die männliche Form verwendet. Gemeint ist stets sowohl die weibliche als auch die männliche Form.

Europa fördert Niedersachsen
EUROPÄISCHE UNION
Europäischer Fonds für regionale Entwicklung

LEUPHANA Inkubator

Ihr persönlicher Studien-Code:
A12345

Einverständniserklärung *(Kopie für Ihre Unterlagen)*

zur Teilnahme an einer wissenschaftlichen Studie

„Unterstützung aus dem Internet: Eine Studie zu Onlineangeboten für Menschen in seelischen Belastungssituationen“

Ich bin mit dem schriftlichen Informationsblatt über die Vorgehensweise der Studie informiert worden.

Teilnahmeerklärung

- Ich entscheide mich **freiwillig und auf eigene Verantwortung** für die Teilnahme an der Studie. Mir ist bekannt, dass keine Patientenversicherung besteht.
- Ich weiß, dass ich meine Zustimmung jederzeit ohne Begründung und ohne Nachteile für mich unter Angabe meines Studien-Codes oder meiner angegebenen E-Mail-Adresse schriftlich widerrufen kann.
- Mir ist bewusst, dass die Studienteilnahme keinen Therapieersatz darstellt und ich weiter medizinische und therapeutische Leistungen in Anspruch nehmen kann.
- Mir wurde empfohlen, meinen Arzt[1] mit dem beiliegenden „Informationsblatt für Ärztinnen und Ärzte“ über meine Studienteilnahme zu informieren und bei einer Zustandsverschlechterung zu kontaktieren.
- Ich weiß, dass per Los entschieden wird, ob ich an „HelpID“ teilnehme oder das Onlineangebot mit Informationstexten zu Gesundheit und Depression erhalte. In letzterem Fall bekomme ich nach Studienende per E-Mail einen Zugang für die Nutzung von „HelpID“.
- Ich bin einwilligungsfähig und habe keinen rechtlicher Betreuer (§ 1896 BGB) oder Bevollmächtigten.

Einwilligungserklärung

- Ich bin einverstanden per Online-Fragebogen Angaben zu meinem Befinden und meiner Zufriedenheit mit den Online-Unterstützungsangeboten zu machen. Die Befragungen werden vor Beginn, sechs Wochen nach Beginn sowie nach Beendigung der Nutzungsdauer und erneut drei und sechs Monate später durchgeführt. Hierzu wird mich das Studienzentrum per E-Mail anschreiben.
- Ich bin einverstanden, dass die von mir erhobenen Daten **pseudonymisiert** werden (durch Zuordnung eines Codes und ohne Nennung meines Namens oder anderer personenbezogener Angaben) und damit keine Rückschlüsse auf meine Person zulassen. Meine Daten werden durch die beteiligten Wissenschaftler ausschließlich zu Studienzwecken gespeichert und ausgewertet. Meine persönlichen Angaben (E-Mail-Adresse und Unterschrift) werden vertraulich behandelt. Ich weiß, dass eine Weitergabe meiner Daten an Dritte ausgeschlossen ist.
- Ich stimme zu, dass die Leuphana Universität Lüneburg die KKH über meine Einverständniserklärung informiert und willige ein, dass die KKH Versicherungsdaten, die meine in Anspruch genommenen medizinischen Leistungen sechs Monate vor und nach meiner Studienteilnahme betreffen, pseudonymisiert an die Leuphana Universität Lüneburg weitergibt, damit diese mit den von mir im Rahmen der Studie erhobenen Daten verknüpft und in die Auswertung einbezogen werden können.

Die gesamte Untersuchung unterliegt den rechtlichen Bestimmungen zum Datenschutz.

Bitte senden Sie das unterschriebene Formular im beiliegenden Rücksendeumschlag an:

Innovations-Inkubator/KT „Vernetzte Versorgung“ Zusatzmodul „Onlinetherapie“
Leuphana Universität Lüneburg
Stichwort: „Online-Studie“
Rotenbleicher Weg 67
21335 Lüneburg

Durch meine Unterschrift erkläre ich mich einverstanden, an der Studie teilzunehmen und bestätige meine Einwilligung in die beschriebene Datenerhebung, -verarbeitung und -nutzung im Rahmen der Studie.

Ort, Datum, Unterschrift:

...

Wichtig! Bitte tragen Sie hier Ihre (möglichst anonyme) E-Mail-Adresse ein:

...

---

[1] Aus Gründen der besseren Lesbarkeit wird im Text nur die männliche Form verwendet. Gemeint ist stets sowohl die weibliche als auch die männliche Form.

Europa fördert Niedersachsen
EUROPÄISCHE UNION
Europäischer Fonds für regionale Entwicklung
