# Supplementary figures and images for: Effectiveness of a Web-Based Intervention in Reducing Depression and Sickness Absence: Randomized Controlled Trial
Source: J Med Internet Res. 2017 Jun 15;19(6):e213. doi: 10.2196/jmir.6546 (PMC5491897; doi:10.2196/jmir.6546)

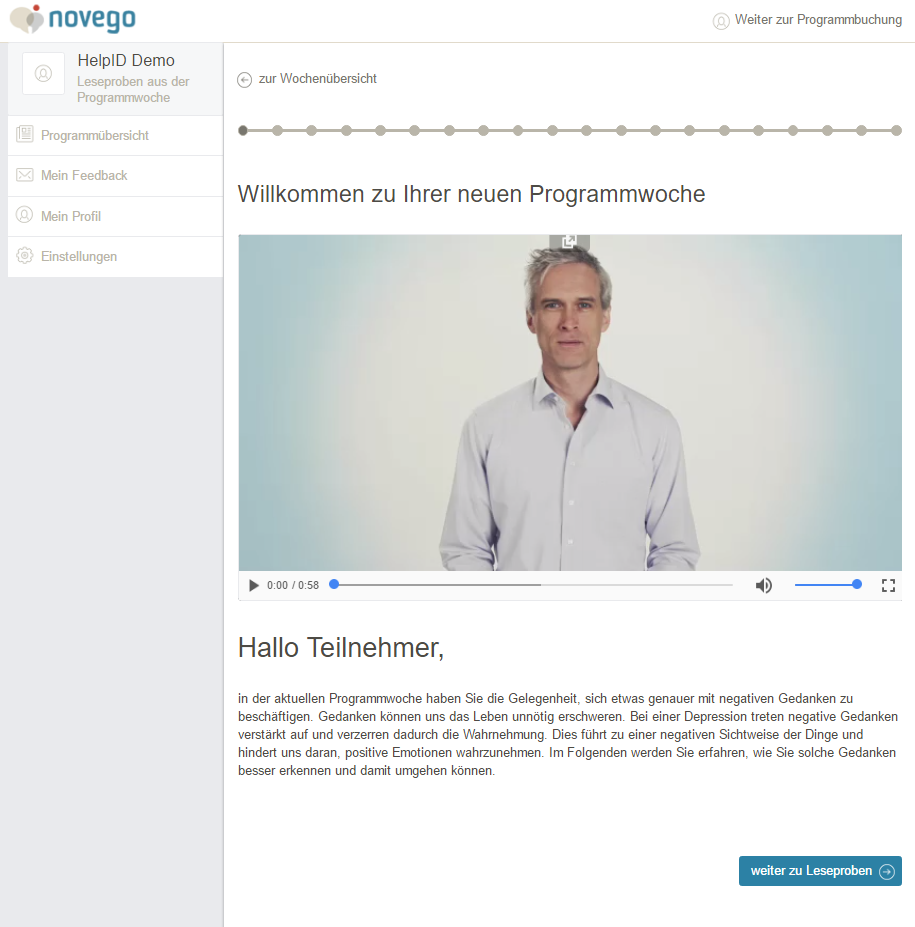

Supplement: Multimedia Appendix 2 [file jmir_v19i6e213_app2.zip › Screenshots/01_HelpID_Demo.PNG]

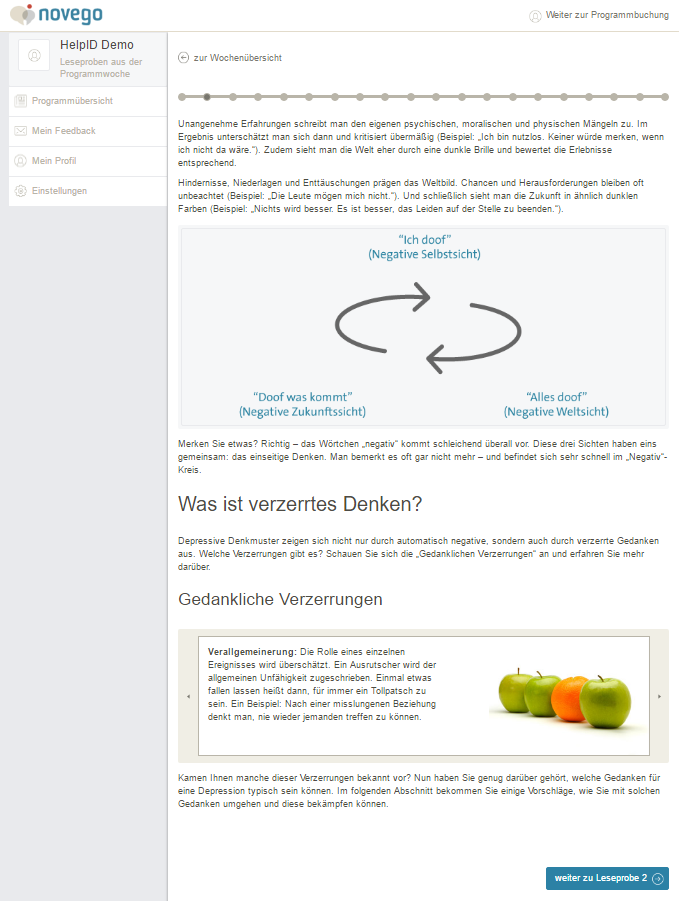

Supplement: Multimedia Appendix 2 [file jmir_v19i6e213_app2.zip › Screenshots/02_HelpID_Demo.PNG]

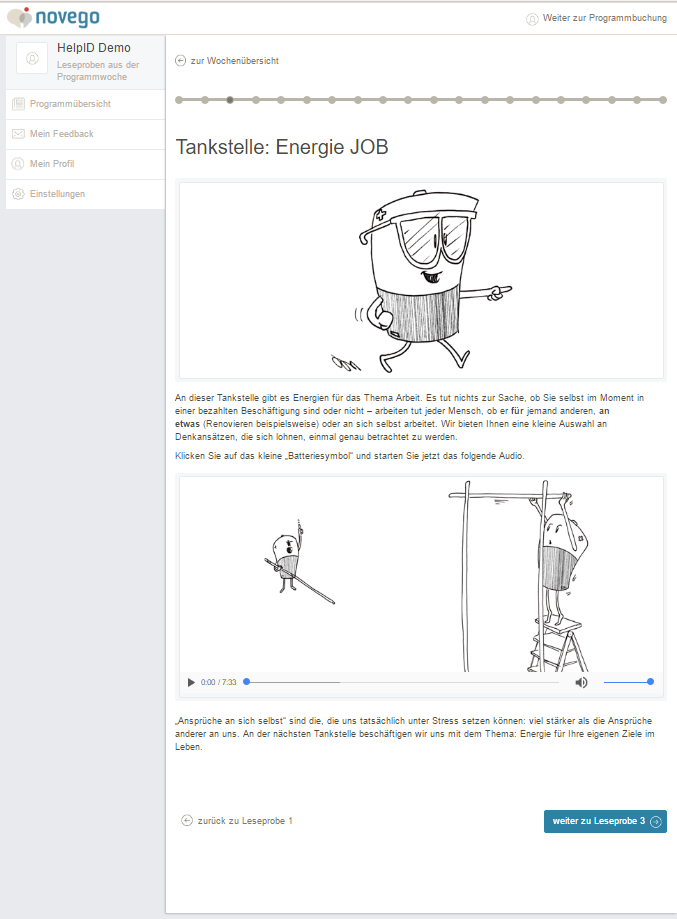

Supplement: Multimedia Appendix 2 [file jmir_v19i6e213_app2.zip › Screenshots/03_HelpID_Demo.PNG]

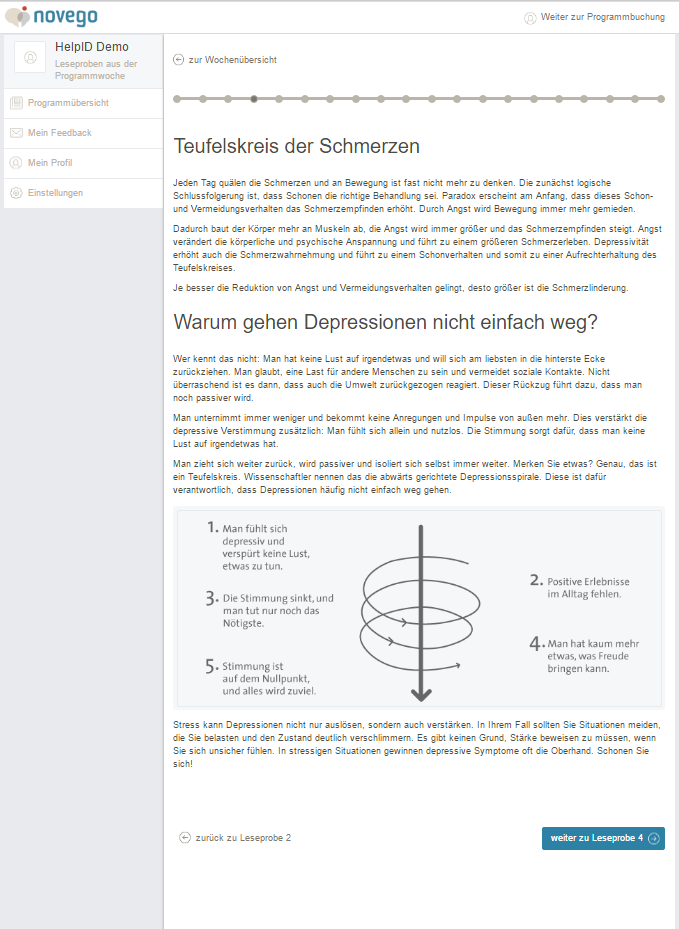

Supplement: Multimedia Appendix 2 [file jmir_v19i6e213_app2.zip › Screenshots/04_HelpID_Demo.PNG]

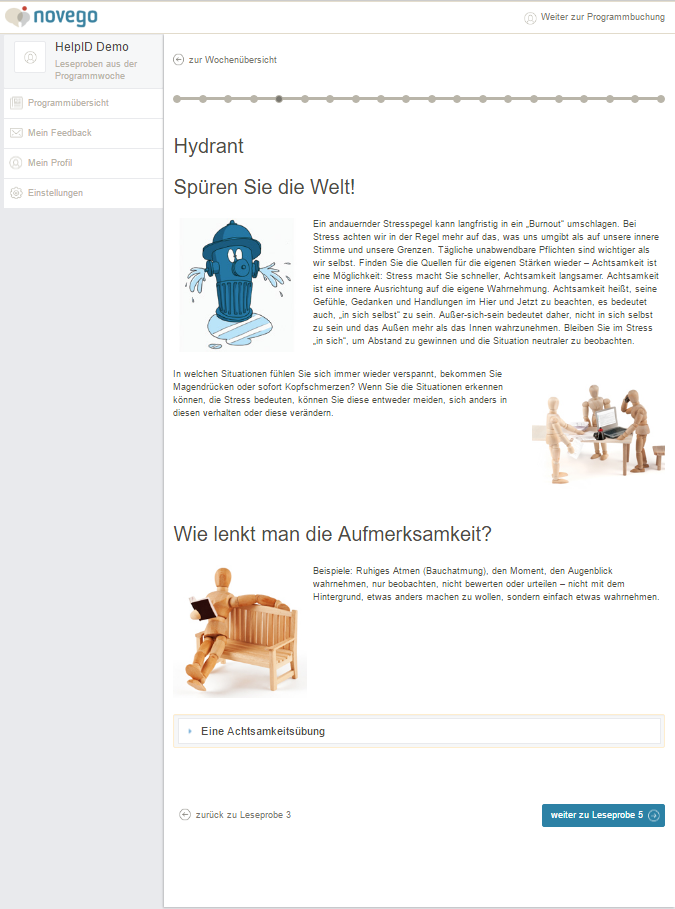

Supplement: Multimedia Appendix 2 [file jmir_v19i6e213_app2.zip › Screenshots/05_HelpID_Demo.PNG]

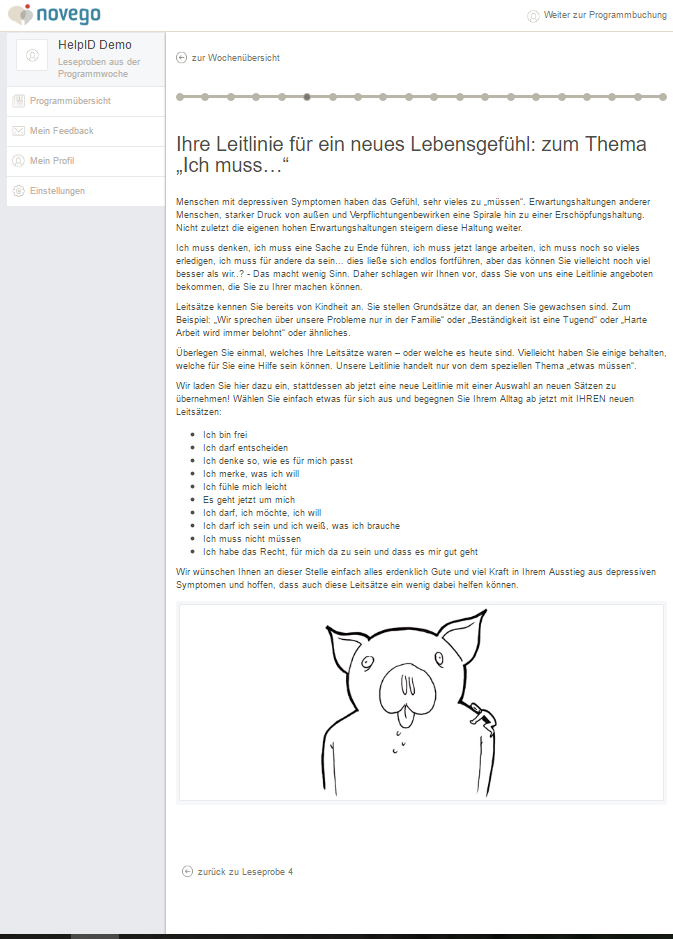

Supplement: Multimedia Appendix 2 [file jmir_v19i6e213_app2.zip › Screenshots/06_HelpID_Demo.PNG]
